# Monocytes secrete CXCL7 to promote breast cancer progression

**DOI:** 10.1038/s41419-021-04231-4

**Published:** 2021-11-17

**Authors:** Yi-Hsiang Wang, Chia-Yi Shen, Sheng-Chieh Lin, Wen-Hung Kuo, Yuan-Ting Kuo, Yu-Ling Hsu, Wen-Ching Wang, Kai-Ti Lin, Lu-Hai Wang

**Affiliations:** 1 Institute of Molecular Medicine, National Tsing Hua University, Hsinchu, Taiwan; 2 Institute of Molecular and Genomic Medicine, National Health Research Institutes, Miaoli, Taiwan; 3 Institute of Biotechnology, National Tsing Hua University, Hsinchu, Taiwan; 4 Graduate Institute of Integrated Medicine and Chinese Medicine Research Center, China Medical University, Taichung City, Taichung Taiwan; 5 Department of Surgery, National Taiwan University Hospital, Taipei, Taiwan; 6 Institute of Molecular and Cellular Biology, National Tsing Hua University, Hsinchu, Taiwan; 7 Department of Life Sciences, National Tsing Hua University, Hsinchu, Taiwan; 8 Department of Medical Sciences, National Tsing Hua University, Hsinchu, Taiwan

**Keywords:** Breast cancer, Cancer microenvironment, Target identification, Chemokines

## Abstract

Certain immune cells and inflammatory cytokines are essential components in the tumor microenvironment to promote breast cancer progression. To identify key immune players in the tumor microenvironment, we applied highly invasive MDA-MB-231 breast cancer cell lines to co-culture with human monocyte THP-1 cells and identified CXCL7 by cytokine array as one of the increasingly secreted cytokines by THP-1 cells. Further investigations indicated that upon co-culturing, breast cancer cells secreted CSF1 to induce expression and release of CXCL7 from monocytes, which in turn acted on cancer cells to promote FAK activation, MMP13 expression, migration, and invasion. In a xenograft mouse model, administration of CXCL7 antibodies significantly reduced abundance of M2 macrophages in tumor microenvironment, as well as decreased tumor growth and distant metastasis. Clinical investigation further suggested that high CXCL7 expression is correlated with breast cancer progression and poor overall survival of patients. Overall, our study unveils an important immune cytokine, CXCL7, which is secreted by tumor infiltrating monocytes, to stimulate cancer cell migration, invasion, and metastasis, contributing to the promotion of breast cancer progression.

## Introduction

Breast cancer is the most commonly occurring cancer and the leading cause of cancer-related death in women worldwide [1]. Due to metastasis and resistance to systemic therapy, the mortality of breast cancer accounts for 15.5% of cancer-related death in females [1]. In recent years, the landscape of tumor microenvironment (TME), especially tumor infiltrating immune cells and cytokines, has been recognized as key factors in affecting cancer progression [2]. Targeting TME, particularly for molecules related to breast cancer metastasis, should be considered for future development of breast cancer therapy.

Macrophages are important immune components in all tissues, where they play important roles in innate immunity [3]. Among the various immune cells present in the TME, tumor-associated macrophages (TAMs) are the predominant population of tumor-infiltrating immune cells [4]. Presence of TAMs in breast cancer is associated with poor prognosis and correlates with drug resistance [5]. The TAMs in breast cancer are largely derived from bone marrow monocytes that are recruited to the TME through inflammatory cytokines released by cancer cells [6]. After arriving at the TME, monocytes differentiate into macrophages by colony-stimulating factor 1 (CSF1, also known as M-CSF) [7]. In breast cancer, most TAMs will be further polarized into immunosuppressive M2-like macrophages [5] by cytokines such as interleukin (IL)−4, IL-10, and IL-13 [8]. In TME, TAMs promote cancer progression by participating in the tumor growth, angiogenesis, cell invasion, cell survival, and immune suppression [4]. Specifically, TAMs secrete matrix metalloproteinases (MMPs), serine proteases, and cathepsins to disrupt cell–cell junctions facilitating cancer cells intravasation and extravasation [9]. On the other hand, TAMs mediate immunosuppression through either expressing high levels of T-cell immune checkpoint ligands, such as PDL1, PDL2, CD80, and CD86, to directly inhibit T cells, or releasing cytokines which contribute to the maintenance of immunosuppressive TME [10, 11].

Chemokines are important mediators for immune cell trafficking and differentiation [12]. In the TME, different immune cells are recruited and further differentiated via interactions between chemokines and chemokine receptors [13]. Among them, Chemokine (C–X–C motif) ligand 7 (CXCL7; also known as NAP-2), which belongs to CXC chemokine family, is originally identified as a crucial player in neutrophil recruitment upon vascular injury by binding to the CXCR2 receptor [14]. In cancer cells, CXCL7 exerts promotion of cancer development in a variety of different cancer types, including renal cell carcinoma [15], lung cancer [16], and breast cancer [17, 18], although the underlying mechanism, especially its role in metastasis, remains unclear.

Here we identified a novel crosstalk between monocytes and breast cancer cells, in which monocytes secrete CXCL7 in response to CSF1 released from invasive breast cancer cells, and in turn CXCL7 acts on cancer cells. Treatment with recombinant CXCL7 protein enhanced chemotaxis of monocytes and promoted migration and invasion of breast cancer cells through FAK- and MMP13-mediated pathways. Blocking CXCL7 by a neutralizing antibody led to the suppression of tumor growth and distant metastasis in a xenograft mouse model, revealing a therapeutic potential of using CXCL7 antibody for cancer immunotherapy. Overall, our current study revealed a novel regulatory mechanism on the interplay of invasive breast cancer cells and tumor infiltrating monocytes in the TME to promote breast cancer cell growth and metastasis, and CXCL7 may serve as a potential therapeutic target for breast cancer immunotherapy.

## Methods

### Cell culture

Human breast cancer cell lines (MDA-MB-231, Hs578T, BT-549) were cultured in Dulbecco’s Modified Eagle’s Medium (DMEM, ThermoFisher, USA) medium containing 10% Fetal Bovine Serum (FBS, Biological Industries, USA) and 1% Penicillin/Streptomycin (P/S). Mouse breast cancer cell line 4T1 and human monocytic leukemia cell lines (THP-1, U937) were cultured in RPMI1640 (ThermoFisher, USA) medium with 10% FBS and 1% P/S. All cells were purchased from Bioresource and collection and research center (BBRC, Taiwan) or American Type Culture Collection (ATCC, USA) and authenticated by STR before shipping. MDA-MB-231 and IV2 were authenticated by STR before xenograft experiments. Mycoplasma contamination was tested if concerned. Primary bone marrow-derived mouse monocytes were from C57BL/6 mice and isolated by EasySep mouse monocytes isolation kit (Stem Cell Technologies, USA). Mouse monocytes were cultured in RPMI1640 medium containing 10% FBS and 1% P/S. All cells were cultured at 37 °C and 5% CO_2_ with humidity.

### Co-culture system and cytokine array

Co-culture was performed using transwell chamber system (0.4-μm pores, BD Falcon, USA) in 24-well dish, in which 1 × 10^5^ human or mouse breast cancer cells were seeded in the bottom well while 1 × 10^5^ THP-1, U937, or mouse monocytes were seeded in the upper chamber. After 48 h incubation, the media was harvested and centrifuged at 1200 rpm to remove the cell pellet. Human Chemokine Array (C1; RayBiotech, USA) was then applied to detect differential chemokine expressions. The chemokine array experiments were carried out in duplicate.

### Enzyme-linked immunosorbent assay (ELISA assay)

The concentration of human CXCL7 cytokine from the co-cultured media was determined by ELISA kit (RayBiotech, USA). The human CSF1 cytokine was measured using solid-phase sandwich ELISA kit (Invitrogen, USA). All procedures followed the manufacturer’s instruction. Data shown represent the means ± SD (*n* = 3 biological replicates).

### Transwell migration and invasion assays

Cell migration was assayed in Falcon Cell Transwell Inserts (8.0-μm pores, BD Falcon, USA), and for the cell invasion assay, the biocoat matrigel invasion chamber was used (BD Falcon, USA) and performed as previously described [19]. Briefly, 2.5 × 10^4^ MDA-MB-231 or Hs578T cells pre-treated with CXCL7 or pre-co-cultured with THP-1 or U937 cells were suspended in DMEM (300 μL) and seeded on the uncoated upper transwell membrane for migration assay or on matrigel-coated membrane for invasion assay. The bottom well was filled with 500 μL DMEM with 10% FBS. After incubation for 8 h (migration assay) or 18 h (invasion assay), cells on the upper side of the inserts were removed by cotton swabs, and cells adhered on the underside were fixed and stained with crystal violet. Photos of three regions were taken and the numbers of cells were counted using Image J (NIH, US). Data shown represent the normalized means ± SD (*n* = 3 biological replicates).

### Chemotaxis assay

A transwell Falcon Cell Transwell Inserts (8.0-μm pores, BD Falcon, USA) was applied for chemotaxis assay. Briefly, 5 × 10^5^ THP-1 cells were seeded on the upper chamber membrane with 8 μm pores in 24-well. The serum-free media containing different concentrations of recombinant human CXCL7 (10, 20, and 30 ng/ml) were applied in lower chamber for chemotaxis assay. Medium with 10% FBS in lower chamber was considered as the positive control. After 16 h, inserts were removed and cells migrated to the underside of the insert were collected and labeled with 1 μM CellTracker™ Red CMTPX (Invitrogen) at 37 °C for 20 min. Fluorescence intensity was measured at Ex/Em = 540/600 nm in Synergy HTX Multi-Mode Reader (Biotek, USA). The number of cells migrated toward 10% FBS was considered as 100 percent migration. Data shown represent the percentage of migrated cells ± SD (*n* = 3 biological replicates).

### Transfection & reagents

Breast cancer cells were transfected with siRNA (50 nM) by TransIT X2 (Mirus bio, USA). The siRNA sequence is listed in Supplemental Table S1. All the recombinant chemokines and antibodies used in this study were listed in Supplemental Table S2.

### Quantitative RT-PCR analysis

RNAs were extracted from control or treated cells using TRIzol (Invitrogen) following protocols supplied by the manufacturer. First-strand cDNA was generated by ReverTraAce (Toyobo, Japan) using oligo-dT as the primer. Real-time RT-PCR was performed on qTOWER 3 Real-Time PCR Thermal Cyclers (Analytik Jena, Germany). The KAPA SYBR FAST Universal qPCR Kit (KAPA Biosystems, USA) was used. The mRNA levels were normalized to that of actin. All primer sequences used in this study are provided in Supplemental Table S3. Data shown represent the normalized means ± SD (*n* = 3 biological replicates).

### Western blot analysis

Cells were lysed in a 1X RIPA buffer (50 mM Tris buffer, pH 7.4, 150 mM NaCl, 1% Triton X-100, 1 mM EDTA, 0.1% SDS, and protease inhibitor mixture (Roche, USA)). The cell lysates were resolved in a 7.5**–**10% SDS-polyacrylamide gel, transferred onto PVDF membrane (Millipore). After protein transfer, membranes were blocked in 3% BSA-PBST (1x PBS with 0.2% Tween20) buffer at RT 30 min and then probed with indicated primary antibodies. Membranes were washed twice with PBST, then incubated with HRP-linked secondary antibody for 1 h at room temperature. After washes with PBST, ECL reagent (Millipore, USA) was used to capture luminescence by Lumin4000 (GE, USA). All antibodies used are listed in Supplemental Table S2. All experiments were repeated three times, and data shown represent the normalized means ± SE (*n* = 3 biological replicates).

### In vivo xenograft mouse model studies

For CXCL7 antibody treatment analysis, the female SCID mice were randomly assigned to each group after arriving and injected with 1 × 10^6^ MDA-MB-231-IV2 cells in 100 µl PBS containing matrigel per mouse at the 4^th^ mammary fat pad at age of 4 to 8 weeks. No sample size calculation was performed. The sample size was inferred from previous studies. For intra-tumor antibody injection, each mouse tumor was injected with 20 µl of CXCL7 antibody (20 µg) or IgG_1_ control (20 µg) per week. For intra-venous antibody injection, tail vein was injected with 100 µl CXCL7 antibody (50 µg) or IgG_1_ control (50 µg) in PBS twice per week. Mice were measured for body weight and tumor size per week. After 28 days post injection, mice were sacrificed, tumors and organs (lung, axillary lymphoid node, thigh bone marrow) were harvested for RNA extraction to detect metastatic cells. No blinding was done. Relative amounts of metastatic IV2 cells from individual organ were measured by quantitative RT-PCR with human specific GAPDH levels and then normalized with actin control, which is compatible to detect actin mRNAs from both human and mouse. All procedures used in our mouse study were performed according to the approved protocol by the Institutional Animal Care and Use Committee of China Medical University, Taiwan (CMUIACUC-2018-138).

### Immunohistochemistry (IHC) analysis

Mouse breast tumor sections were prepared by pathology core lab service (NHRI), and stained with mouse macrophage marker CD206 (eBioscience, USA) and F4/80 (Abcam, USA) antibodies.

### Clinical samples

Frozen breast tumor samples and adjacent nontumor tissues were obtained from the Department of Surgery, National Taiwan University Hospital (NTHU), under an approved IRB protocol (201505011RINC). Samples were collected during debulking surgery, the identities of the patients remained anonymous, and informed consent was obtained from all subjects. For data from public RNA-seq database, the gene expression profile of CXCL7 and patients’ clinical information was obtained from The Cancer Genome Atlas (TCGA) (https://cancergenome.nih.gov) and Gene Expression Omnibus (GEO).

### Kaplan–Meier plotter database analysis

Kaplan–Meier Plotter (http://kmplot.com/analysis/) is an online database of published RNAseq array datasets that assesses the effect of 54,675 genes on survival. We performed a Kaplan–Meier Plotter analysis to assess the prognostic value of CXCL7 in patients with breast cancer or other cancer types. The hazard ratios (HRs) with 95% confidence intervals (CIs) and log-rank *p*-values were also computed.

### Statistical method

GraphPad Prism software was used for statistical analysis (GraphPad Software, Inc.). The results were shown with mean ± SD or ± SEM from three biological replicates. Student’s *t* test two-tail statistics analysis was used to compare two means. ANOVA followed by Tukey’s post hoc test was used for the statistical analysis when more than two means were compared. The significant *p* value was showed in star(s). (*, *p* < 0.05; **, *p* < 0.01, ***, *p* < 0.001, ****, *p* < 0.0001).

## Results

### Identification of CXCL7 cytokine in the co-cultured media of monocytes and invasive breast cancer cells

**Fig. 1:**
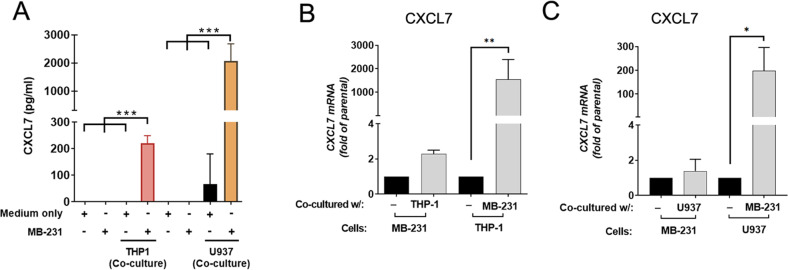
MDA-MB-231 breast cancer cells induce CXCL7 secretion from THP-1 and U937 monocytes. **A** The CXCL7 protein concentration in cultured media from medium only, medium from MDA-MB-231 cells, THP-1 or U937 monocytes, or MDA-MB-231 cells co-cultured with THP-1 or U937 monocytes for 48 h, were determined by ELISA assay. Data represent the means ± SD (*n* = 3 biological replicates; ***, *p* < 0.001). **B**, **C** CXCL7 mRNA expression in MDA-MB-231 cells co-cultured with THP-1 (**B**), or U937 (**C**) monocytes for 48 h. RNA was extracted from either MDA-MB-231, THP-1, or U937 cells as indicated. Data represent the normalized means ± SD (*n* = 3 biological replicates, *p < 0.05; **p < 0.01).

To identify important cytokines released in the TME that promote breast cancer metastasis, the highly invasive breast cancer MDA-MB-231 cells were subjected to the co-culturing system with THP-1 monocytes. In this system, MDA-MB-231 cells were seeded on the bottom of a 6-well plate overlaid with a transwell insert seeded with THP-1 monocytes without direct contact but were cultivated in the same culture media for 48 h. After incubation, the co-cultured media (CM) was collected and subjected to cytokine array analysis. CM from MDA-MB-231/THP-1 showed upregulation of C–X–C motif ligand (CXCL) 1, 7, 8, 10, and C–C motif chemokine ligand (CCL) 2–5, as compared to the cultured media from THP-1 or MDA-MB-231 alone (Fig. S1A, B). Notably, CXCL7 was the only CXCL cytokine found in the CM from THP-1 monocytes (Fig. S1A), suggesting CXCL7 may be secreted by THP-1 cells and played a role in the crosstalk between monocytes and invasive breast cancer cells in the TME. We further validated that CM from MDA-MB-231 cells co-cultured with THP-1 or U937 monocytes showed significant higher levels of secreted CXCL7 proteins by ELISA assay (Fig. 1A). By analyzing mRNA expression in MDA-MB-231 breast cancer cells and in monocytes (THP-1 or U937), respectively, after co-culturing for 48 h, CXCL7 levels were found to be strongly enhanced in the THP-1 or U937 monocytes co-cultured with MDA-MB-231 cells (Fig. 1B, C), and the same phenomenon was observed upon co-culturing of the monocytes with another breast cancer cell line, Hs578T cells (Fig. S1C). On the other hand, there was no significant induction of CXCL7 expression in THP-1 monocytes when co-cultured with BT-549 cells (Fig. S1D), suggesting not all breast cancer cell lines could stimulate CXCL7 expression and secretion in monocytes. To further inquire whether CXCL7 could be induced from normal monocytes upon co-culturing with the breast cancer cells, we harvested mouse monocytes from bone marrow and subject to co-culturing with invasive mouse breast cancer cells, 4T1. After incubating for 48 h, CXCL7 levels were significantly enhanced in the mouse monocytes co-cultured with 4T1 cells (Fig. S1E). Overall, our data indicate that monocytes express and secrete CXCL7 in response to tumor cells in the TME.

### CSF1 released by breast cancer cells induces CXCL7 secretion by monocytes

**Fig. 2:**
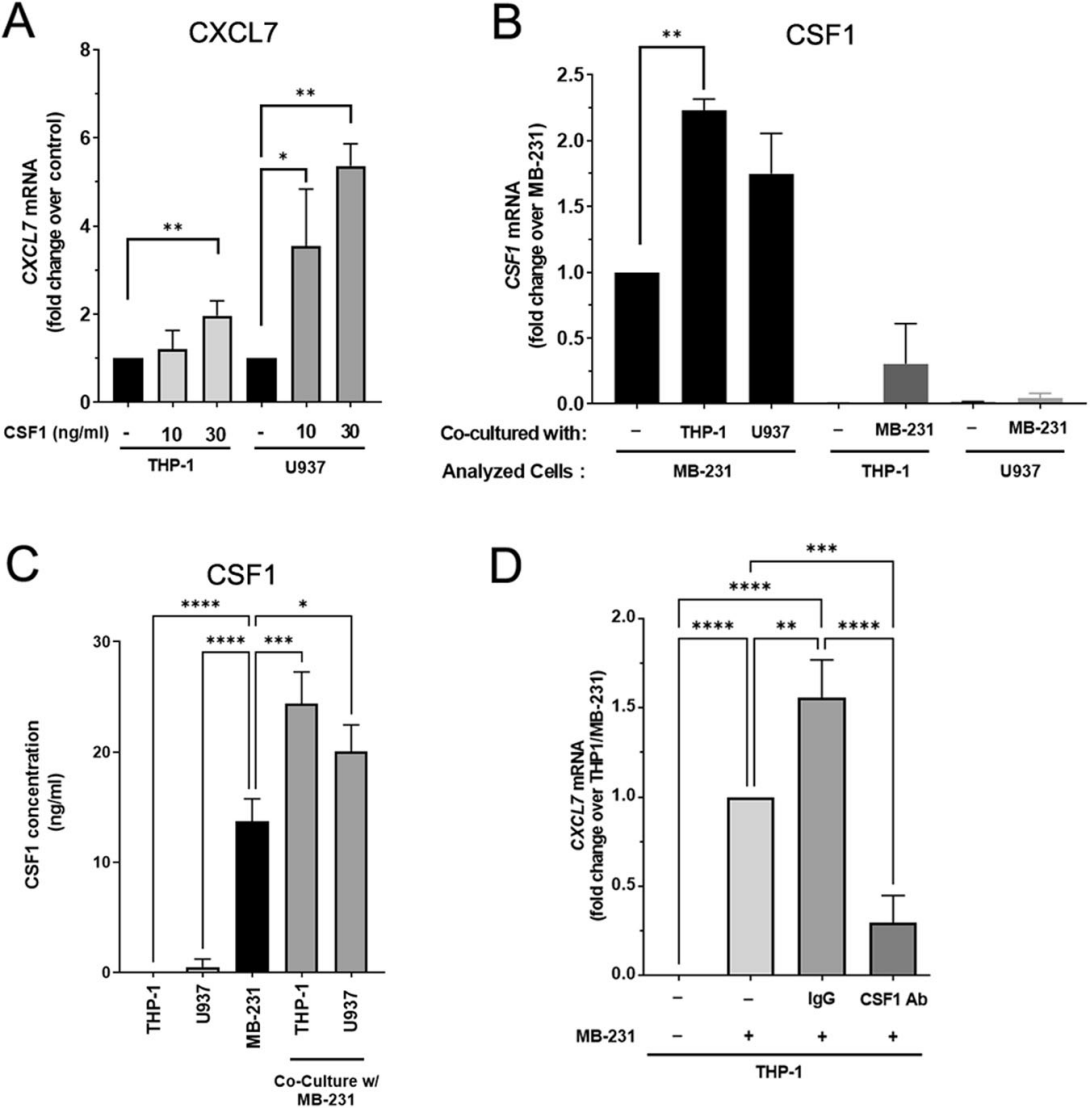
CSF1 secreted by MDA-MB-231 cells induces CXCL7 expression from THP-1 monocytes. **A** CXCL7 mRNA expression in the presence of recombinant CSF1. THP-1 or U937 cells were treated with recombinant CSF1 (10 or 30 ng/mL) for 48 h, RNA was then extracted to detect CXCL7 expression. Data represent the normalized means ± SD (*n* = 3 biological replicates; *p < 0.05; **p < 0.01). **B** CSF1 mRNA expression in MDA-MB-231, THP-1, U937 cells, or MDA-MB-231 cells co-cultured with THP-1 or U937 monocytes for 48 h. RNA was extracted from MDA-MB-231, THP-1, or U937 cells for analysis as indicated. Data represent the normalized means ± SD (*n* = 3 biological replicates; **p < 0.01). **C** The CSF1 protein concentration collected from the cultured media of MDA-MB-231, THP-1, U937 cells only or MDA-MB-231 co-cultured with THP-1 or U937 cells for 48 h were determined by ELISA assay. Data represent the means ± SD (*n* = 3 biological replicates; *p < 0.05; ***p < 0.001; ****p < 0.0001). **D** CXCL7 mRNA expression in THP-1 cells co-cultured with MDA-MB-231 cells in the presence of CSF1 neutralizing antibodies (5 μg/ml) or equivalent amount of control IgG for 48 h, RNA was extracted to detect CXCL7 expression. The level of CXCL7 mRNA in MDA-MB-231 cells co-cultured with THP-1 monocytes was used as a control. Data represent the normalized means ± SD (*n* = 3 biological replicates; **p < 0.01; ***p < 0.001; ****p < 0.0001).

Monocytic recruitment to the TME is often mediated by tumor-secreted soluble factors, such as CSF1 and CCL2 [4]. We, therefore, attempted to examine whether tumor-derived CSF1 or CCL2 was responsible for CXCL7 secretion. The level of CXCL7 was significantly increased in both THP-1 and U937 monocytes in the presence of CSF1 (Fig. 2A). In contrast, CCL2 only stimulated CXCL7 expression in THP-1, but not in U937 (Fig. S2). Since CSF1 could stimulate CXCL7 expression in both monocytic cell lines, we decided to focus on CSF1 first to determine whether it could stimulate monocytes to express and secrete CXCL7 to the TME. Both mRNA and protein expression of CSF1 was higher in MDA-MB-231 cells than that in THP-1 or U937 monocytes, and elevated CSF1 expression was observed in MDA-MB-231 cells upon culturing with THP-1 or U937 monocytes (Fig. 2B, C). Treatment with neutralizing antibodies against CSF1 in the co-culture system significantly blocked CXCL7 expression in THP-1 monocytes (Fig. 2D). In summary, our data indicate that breast cancer cells secrete CSF1 to induce monocytic expression and secretion of CXCL7 in the TME.

### CXCL7 promotes breast cancer cell migration and invasion through FAK-and MMP13-mediated signaling pathway

**Fig. 3:**
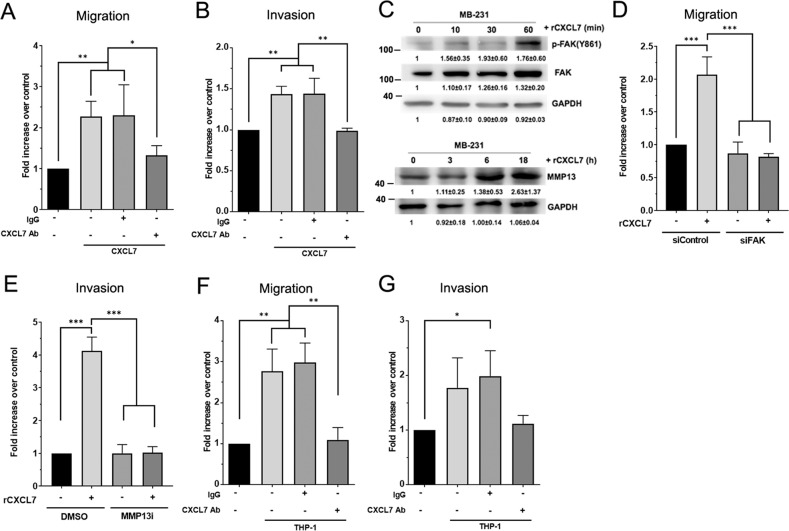
Monocyte-derived CXCL7 induces breast cancer cell migration and invasion via FAK- and MMP13-mediated signaling pathways. **A**, **B** Migration and invasion abilities of MDA-MB-231 cells pre-treated with the recombinant CXCL7 protein (rCXCL7, 5 ng/ml), together with a CXCL7 neutralizing antibody (5 μg/ml) or equivalent amount of a control IgG for 48 h. Cells were then incubated for migration (**A**) and invasion (**B**) assays. Data represent the normalized means ± SD (*n* = 3 biological replicates; *p < 0.05; **p < 0.01). **C** Western blot analysis of phospho-FAK (Y861), FAK, and MMP13 in the MDA-MB-231 cells treated with rCXCL7 (2 ng/ml) in different time points as indicated. GAPDH was used as the internal control. Histograms represent normalized means ± SD (*n* = 3 biological replicates). **D** Migration abilities of MDA-MB-231 cells transfected with Control or FAK siRNA. After transfection, cells were treated with rCXCL7 protein (5 ng/mL) for 48 h and then subjected for migration assay. Data represent the means ± SD (*n* = 3 biological triplicates; ***p < 0.001). **E** Invasion abilities of MDA-MB-231 cells pre-treated with rCXCL7 protein (5 ng/mL) and a MMP13 inhibitor (20 nM). Cells were treated with rCXCL7 and MMP13 inhibitor (MMP13i) for 48 h and then subjected for invasion assay. Data represent the means ± SD (*n* = 3 biological triplicates; ***p < 0.001). **F**, **G** Migration and invasion abilities of MDA-MB-231 cells co-cultured with THP-1, and in the presence of a CXCL7 neutralizing antibody (5ug/ml) or control IgG_1_ for 48 h, cells were then subjected to migration (**F**) and invasion (**G**) assays. Data represent the means ± SD (*n* = 3 biological replicates; *p < 0.05; **p < 0.01).

As described above, we identified CXCL7 as a potential player to promote breast cancer metastasis through the monocyte co-culturing system. To further assess the effects of CXCL7 on cellular function relevant to metastatic behavior, the transwell cell migration and invasion assays were applied. Cell migration assay was performed by transwell Boyden chambers, while cell invasion assay was performed by transwell Boyden chambers pre-coated with matrigel to mimic invasion through the basement membrane. Treatment of recombinant CXCL7 protein enhanced cell migration/invasion in MDA-MB-231 (Fig. 3A, B) and Hs578T breast cancer cells (Fig. S3A), while co-treatment with antibodies against CXCL7 largely reversed the induction of migration/invasion by CXCL7 in MDA-MB-231 cells (Fig. 3A, B). Previous studies showed that CXCR2, the receptor of CXCL7, promoted cell motility through stimulating focal adhesion kinase (FAK)-mediated signaling pathway [20], resulting in the increased expression level of metalloproteinase-13 (MMP-13) [21]. Consistent with their observations, we found that after CXCL7 treatment, the level of activated FAK (pY861) and MMP-13 were significantly increased (Fig. 3C). Knockdown FAK by siRNA significantly decreased CXCL7-promoted migration (Fig. 3D) while treatment with the MMP-13 inhibitor significantly suppressed CXCL7-induced cell invasion in MDA-MB-231 cells (Fig. 3E), suggesting that CXCL7 promoted breast cancer migration/invasion through FAK- and MMP13-mediated signaling pathways.

Next, to confirm that CXCL7 contributed to the monocyte-induced breast cancer cell migration and invasion, we added neutralizing antibodies against CXCL7 to the co-culturing system. Increased migration and invasion abilities were observed in MDA-MB-231 cells co-cultured with THP-1 (Fig. 3F, G) or U937 (Fig. S3B, C) monocytes, and this promotion was blocked by antibodies against CXCL7 (Figs. 3F, G, S3B, C). These results indicate that monocytes derived CXCL7 is sufficient and required to stimulate migration and invasion of breast cancer cells.

### Inhibition of CXCL7 suppresses tumor growth and reduces the incidence of distant metastases in xenograft breast cancer model

**Fig. 4:**
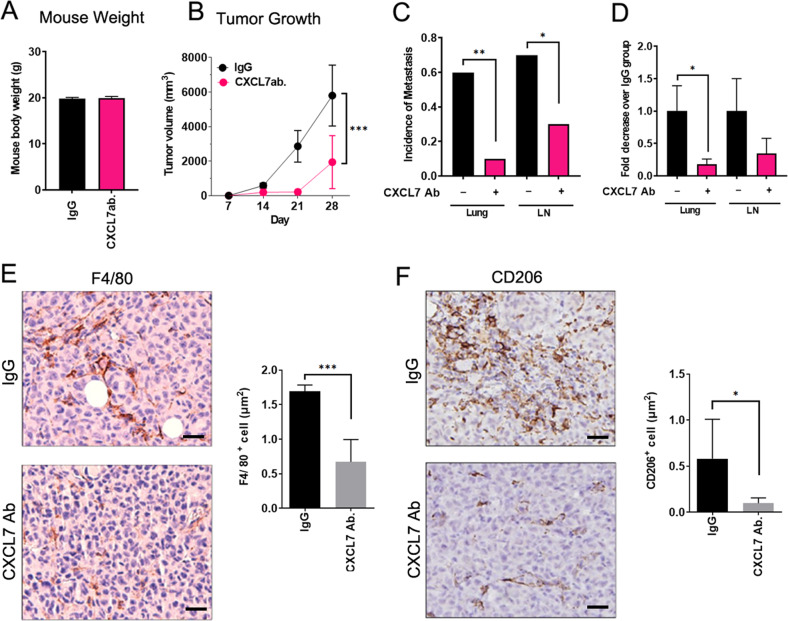
Blocking CXCL7 by a neutralizing antibody suppresses tumor growth and distant metastasis in a mouse xenograft model. 1 × 10^6^ IV2 cells were orthotopically injected into the 4^th^ mammary fat pad per mouse. Seven days after implantation, intratumoral injection of a CXCL7 antibody (1 mg/kg) or equivalent amount of control IgG_1_ was started once a week until the endpoint. **A**–**D** The mouse weight (**A**) and the tumor growth curve (**B**). Data are means ± SEM (*n* = 9 or 10 mice per group; data were combined from two independent experiments). Two-way ANOVA was used for the statistical analysis (*p* < 0.001). The incidence of metastasis (**C**) and relative amounts of metastatic cells (**D**) from individual lung or axillary lymph nodes (LN) were determined and measured by human specific GAPDH levels to quantify relative number of metastatic IV2 cells. Data are normalized to the means of the IgG_1_ control group ± SEM (*n* = 9 or 10 mice per group; data were combined from two independent experiments; *p < 0.05; **p < 0.01). **E**, **F** Immunohistochemical analysis of paraffin-embedded xenograft breast tumors using F4/80 (**E**) and CD206 (**F**) antibodies. The number of positive labeled cells per μm^2^ was quantified. Scale bars: 20 μm. Data are presented as means ± SEM (*n* = 5 mice per group; *p < 0.05; ***p < 0.001).

To test the role of CXCL7 during breast cancer progression, we established the xenograft mouse model by pre-implantation of the MDA-MB-231-IV2 (IV2) cells in the mammary fat pad. IV2 is a highly invasive breast cancer cell line derived from MDA-MB-231 cells through in vivo selection from lung metastasized MDA-MB-231 cells as we reported before [22]. We first confirmed CXCL7 secretion was significantly increased in THP-1 and U937 cells upon co-culturing with IV2 cells in both mRNA and protein levels (Fig. S4A-C). Since IV2 was derived from human breast cancer cell line, MDA-MB-231, we further examined whether human CSF1 (hCSF1) can stimulate mouse CXCL7 (mCXCL7) secretion in bone marrow-derived mouse monocytes. The level of mCXCL7 mRNA was significantly stimulated by hCSF1 in primary mouse monocytes (Fig. S4D), supporting the notion that the crosstalk could be established between IV2 cells and mouse monocytes in the xenograft mouse model. After inoculation of IV2 cells into the mammary fat pad, we started intratumoral injection of the CXCL7 neutralizing antibody once every week for 35 days. The mouse body weight remains similar in different treatment group (Fig. 4A), while tumor growth was greatly inhibited in the CXCL7 antibody, as compared to the control IgG_1_-treated group (Fig. 4B). We then checked the incidence of distant metastases by detecting human specific-GAPDH (hGAPDH) levels through real-time RT-PCR. Metastatic incidence in lung and lymph nodes (LN) were detectable in about 60 and 70%, respectively, of mice in the IgG_1_ control group compared with less than 10 and 30%, respectively, in the group with CXCL7 antibody injection (Fig. 4C), and the relative amount of metastatic IV2 cells were greatly decreased in tumors with CXCL7 antibody injection (Fig. 4D). Similar results were obtained in the same mouse model with intravenous injection of CXCL7 antibodies (Fig. S4E–G), revealing the therapeutic potential of using neutralizing antibodies against CXCL7 to treat breast cancer metastasis.

We next investigated whether inhibition of CXCL7 could affect infiltration of macrophages to the TME in vivo. By analyzing the expression of macrophage marker (F4/80) and M2-type macrophage marker (CD206) in the IV2 xenograft tumors, we found that compared to the IgG_1_ control group, tumors with CXCL7 antibody injection displayed significantly reduced number of F4/80 or CD206 positively labeled cells (Fig. 4E, F), demonstrating that CXCL7 could act as an important modulator to promote breast cancer progression through promoting the recruitment of TAMs. This finding is in agreement with the report showing Lewis lung carcinoma cells overexpressing CXCL7 increased the infiltration of M2 macrophages at the early stages of lung tumorigenesis [16]. In vitro chemotaxis study further confirmed that treatment with CXCL7 increased THP-1 monocytes recruitment (Fig. S4H), reflecting the possibility that release of CXCL7 by tumor infiltrating monocytes stimulates recruitment of circulating myeloid cells into the TME and these recruited myeloid cells would further differentiate into TAMs.

### Expression of CXCL7 correlates with breast cancer progression or patients’ survival

**Fig. 5:**
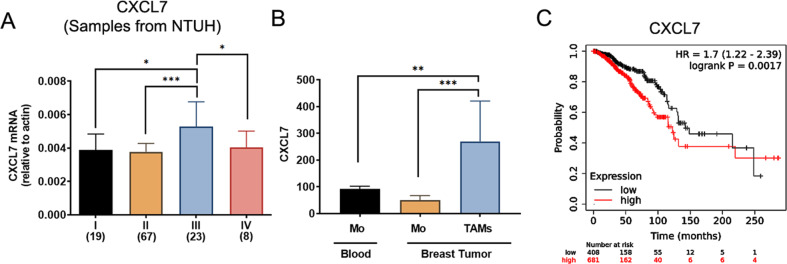
CXCL7 level is elevated in stage III breast carcinoma and correlates with poor survival. **A** Expression of CXCL7 in 117 breast carcinoma samples from NTUH was determined by real-time RT-PCR analysis. Samples were sub-grouped by TNM stages. The case number of each stage is indicated at the bottom of the graph. Data represent the means ± SEM. (*p < 0.05; ***p < 0.001). **B** The mRNA expression of CXCL7 in circulating human monocytes (*n* = 61), human monocytes from breast tumor (*n* = 23), and human tumor associated macrophages from breast tumor (*n* = 4). RNAseq data were obtained from publicly available dataset (GSE117970). Data represent the means ± SEM (**p < 0.01; ***p < 0.001). **C** Kaplan–Meier plots showing the association of CXCL7 expression with overall survival (OS) of breast cancer patients from KM plotter breast cancer sample RNA-seq datasets. The statistical significance was determined using the χ^2^ test.

To evaluate the clinical relevance of CXCL7 in breast cancer progression, we analyzed its expression by real-time RT-PCR in clinical samples of breast cancer carcinoma from National Taiwan University Hospital (NTUH). The expression of CXCL7 was low among all the breast tumor samples we tested and slightly increased in late stage (stage III; Fig. 5A). By analyzing data from TCGA dataset, in agreement with the results of our patient cohort, the CXCL expression was modestly upregulated at late stage of breast cancer (Fig. S5A). Since our data suggested that CXCL7 could be secreted by cancer-associated monocytes (Fig. 1), we further analyzed the publicly available dataset (GSE117970) regarding the CXCL7 expression in monocytes and TAMs. Interestingly, we found that expression of CXCL7 was significantly upregulated in TAMs from breast tumors, as compared to monocytes from circulation blood or breast tumor (Fig. 5B), confirming that CXCL7 is highly expressed in TAMs upon crosstalk with breast tumor cells. To explore the clinical significance of CXCL7, we analyzed the correlation of CXCL7 expression with overall survival from the breast cancer RNA-seq datasets. Kaplan Meier survival analysis revealed that high CXCL7 expression were negatively correlated with overall survival (OS) of breast cancer patients (Fig. 5C), as well as patients of other cancer types (Fig. S5B–E). Overall, our data show that the expression of CXCL7 is significantly correlated with clinical outcome of breast cancer patients, and CXCL7 may serve as a useful prognosis marker for breast cancer patients.

## Discussion

**Fig. 6:**
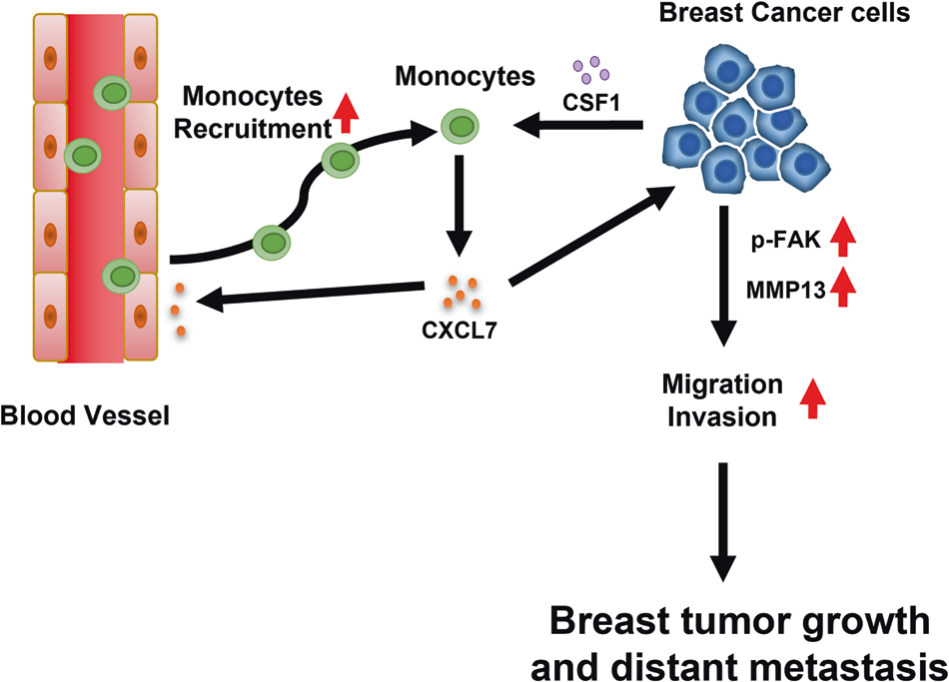
Current working model of the crosstalk between breast cancer cells and monocytes in the TME. Breast cancer cells secrete CSF1 to stimulate monocytes to secrete CXCL7. CXCL7 subsequently recruits monocytes into the TME and enhances invasive behavior in breast cancer cells, resulting in the promotion of breast tumor growth and distant metastasis.

High infiltration of monocyte-derived macrophages in the TME is generally associated with poor clinical outcomes in patients with breast cancer [23]. TAMs contribute to cancer progression at a variety of different levels, including promoting immune suppression, nurturing cancer stem cells, inducing angiogenesis, and paving the way for distant metastasis [4]. Considering TME is evolved together by cancer cells and macrophages during cancer progression, disrupting their interplays in the TME can be a useful strategy to suppress cancer progression. Here we identified a novel crosstalk mechanism between breast cancer cells and monocytes that promotes the recruitment of monocytes and enhances breast cancer cells migration and invasion, resulting in the progression of tumor growth and metastasis (Fig. 6). Moreover, using neutralizing antibodies against CXCL7, we have successfully shown inhibition of both tumor growth and distant metastasis (Figs. 4, S4), revealing a potential therapeutic opportunity of using CXCL7 antibodies for treating metastatic breast cancer.

Most macrophages in the TMEs are originated from bone marrow-derived monocytes that are recruited through inflammatory signals released by cancer cells [24]. Among them, CSF1 is crucial for the differentiation and survival of macrophages [25], and elevated expression of CSF1 correlates with high grade and poor prognosis in breast cancer [26]. CSF1 or CSF1 receptor (CSF1R) are popular therapeutic targets and have been studied in multiple clinical trials to deplete TAMs in the TME [4]. Here our data reveal that CSF1, secreted by breast cancer cells stimulated CXCL7 expression from monocytes to promote monocyte recruitments (Fig. S4H), and to enhance cancer cell migration and invasion (Fig. 3). Our findings shed a new light on our understanding of how CSF1 modulates TME to promote breast cancer progression through CXCL7-mediated signaling pathway. In fact, depletion of macrophages indiscriminately by drugs targeting CSF1 or CSF1R will cause substantial toxicity to normal tissues [4], instead, targeting downstream pro-tumor molecule mediated by CSF1, such as CXCL7, may be a better candidate for future drug development.

Chemokines and chemokine receptors are important regulators to mediate cross-talk between tumor cells and immune cells in the TME [13]. Previous studies mainly focused on the role of these chemokines during cancer development. Nevertheless, the mechanism of releasing these chemokines has been less studied. Our study here reveals that in the co-cultured system, breast cancer cells release molecules to stimulate monocytes to express and release CXCL7 (Fig. 1). We next investigated what molecule(s) secreted by breast cancer cells serves the major role to induce CXCL7 expression. We found CSF1, which was secreted by breast cancer cells (Fig. 2B, C), could directly stimulate CXCL7 expression in monocytes (Fig. 2A). Meanwhile, blocking CSF1 by neutralizing antibodies significantly inhibited CXCL7 expression induced by co-culturing (Fig. 2D), suggesting that CSF1 is an essential regulator for CXCL7 expression. However, the induction of CXCL7 by recombinant CSF1 alone was only 2–6 folds compared with untreated control, which is much less than the hundred folds induction of the mRNA we observed in monocytes co-culturing with breast cancer cells (Fig. 1B, C). It is possible that CSF1 may function in concert with other players, which could also be secreted by breast cancer cells, to work together to stimulate CXCL7 expression robustly. For example, CCL2, which also stimulated CXCL7 expression in THP-1, but not in U937 though (Fig. S2). Another possibility is that the recombinant CSF1 protein we used in this study is derived from bacterial expression system, which may not have optimal post-translational modification for its activity. CSF1 is produced and secreted as a glycosylated protein [27, 28], and the recombinant protein from bacteria may not have the correct protein folding and post-translational modification, such as glycosylation [29]. As a result, the recombinant CSF1 may possess lower activities than the endogenously produced CSF1 from breast cancer cells reflected in Fig. 1 assay.

In this study, clinical samples of breast cancer patients from NTU hospital and publicly available datasets were applied to analyze the expression of CXCL7 and its correlation with the pathological stages and survival outcome. We observed that expression of CXCL7 is very low in the tumor specimen (Figs. 5A, S5A), and the level of CXCL7 was undetectable in half of the samples from the TCGA dataset (Fig. S5A), revealing the difficulties to validate chemokines secreted by monocytes/macrophages in tumor tissues. Despite this, we found CXCL7 level was significantly upregulated in human TAMs in breast cancer by using publicly available dataset of RNA-seq from purified human circulating monocytes and TAMs in breast cancers (Fig. 5B), confirming our suspicion that the levels of macrophage-derived CXCL7 could easily be underestimated in tumor samples. Injection of blocking antibodies against CXCL7 in the xenograft mouse model further suggested CXCL7 was essential for the recruitment of TAMs (Fig. 4E), especially M2-like TAMs (Fig. 4F) in the TME. Overall, our study identifies a novel crosstalk mechanism between breast cancer cells and monocytes to facilitate breast cancer progression and metastasis through CSF1/CXCL7 axis. CXCL7 may be an applicable therapeutic target for breast cancer therapy.

## Supplementary information

SUPPLEMENTAL MATERIAL

## Data Availability

The data presented in this manuscript are available upon reasonable request from the corresponding authors.
