# Supplementary material for: Monocytes secrete CXCL7 to promote breast cancer progression
Source: Cell Death Dis. 2021 Nov 17;12(12):1090. doi: 10.1038/s41419-021-04231-4 (PMC8599470; doi:10.1038/s41419-021-04231-4)
Supplement: Supplementary file 1 — SUPPLEMENTAL MATERIAL [file 41419_2021_4231_MOESM1_ESM.docx]

**Supplemental figure legends**

**
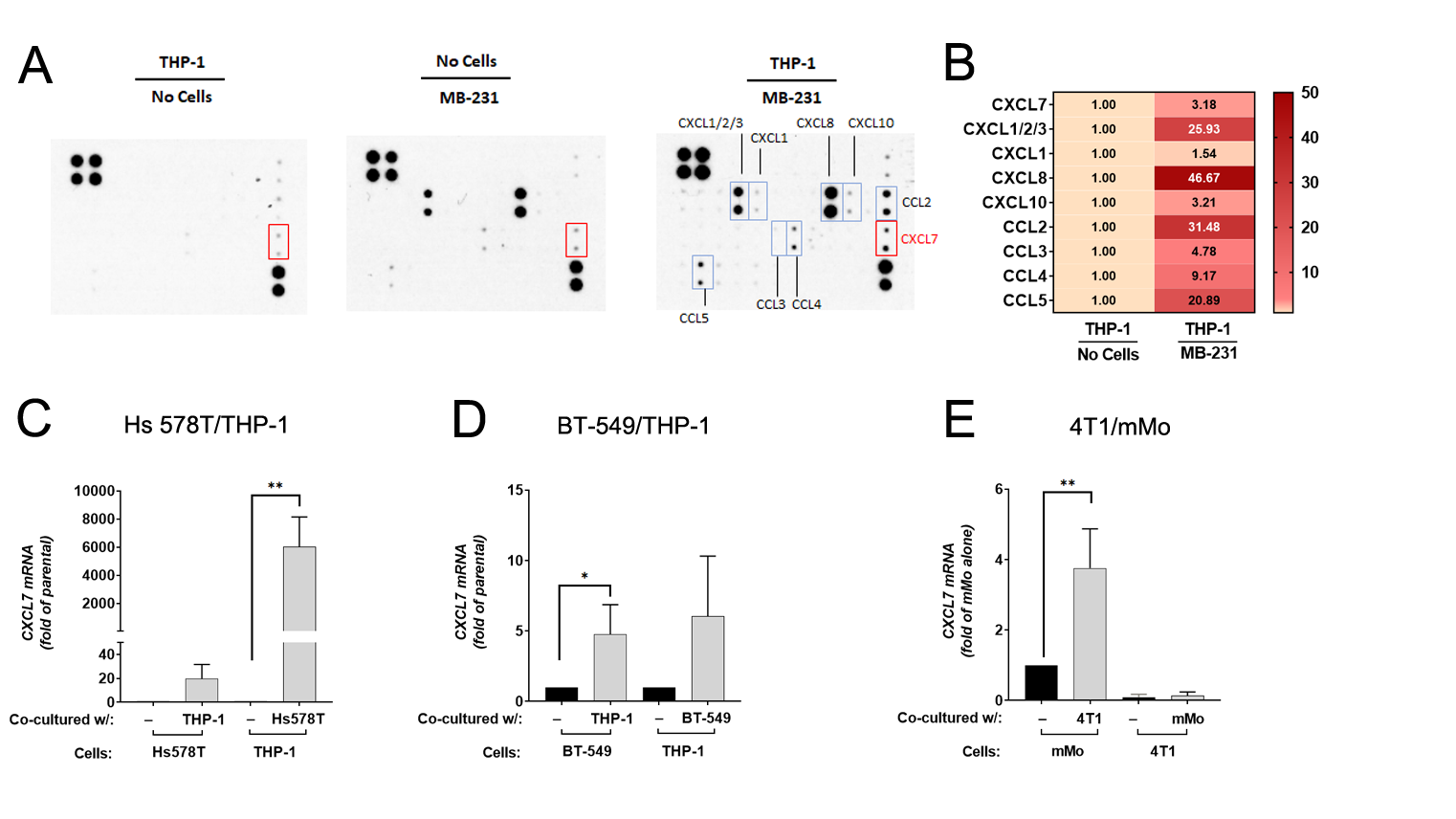
**

**Supplementary figure 1. CXCL7 is secreted by THP-1 monocytes by co-culturing with breast cancer cells.**

(A) Image of chemokine array analysis of conditioned media harvested from co-culture system using human monocytic cell lines, THP-1, co-cultured with MDA-MB-231 breast cancer cells (MB-231) for 48 h.

(B) Heatmap of chemokine profiles obtained from the chemokine array (A). THP-1 alone or THP-1 co-cultured MDA-MB-231 cells for 48 h. Data represent the fold change relative to the THP-1 control.

(C, D) CXCL7 mRNA expression in Hs578T cells (A) or BT-549 cells (B) co-cultured with THP-1 cells for 48 h. Data represent the normalized means ± SD (n= 3 biological replicates, *, p<0.05; **, p<0.01).

(E) CXCL7 mRNA expression in mouse monocytes (mMo) co-cultured with 4T1 breast cancer cells, or in 4T1 cells co-cultured with mouse monocytes for 48 h. Data represent the normalized means ± SD (n= 3 biological replicates, **, p<0.01).

**
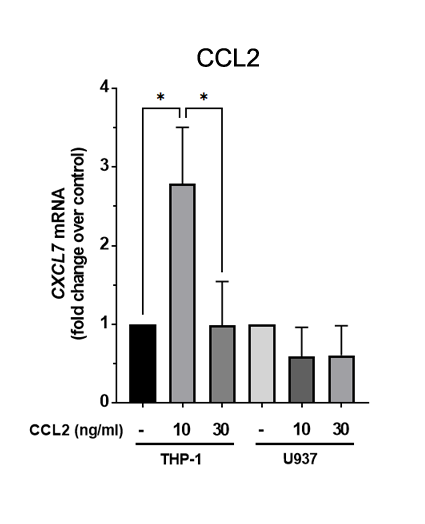
**

**Supplementary Figure 2. CCL2 fails to induce CXCL7 expression in THP-1 and U937 monocytes.**

CXCL7 mRNA expression in the presence of recombinant CCL2. THP1 or U937 cells were treated with recombinant CCL2 (10-30ng/mL) for 48 h, and then RNA was extracted to detect CXCL7 expression. Data represent the normalized means $\pm$SD (n=3 biological replicates; *, *p*<0.05)

**
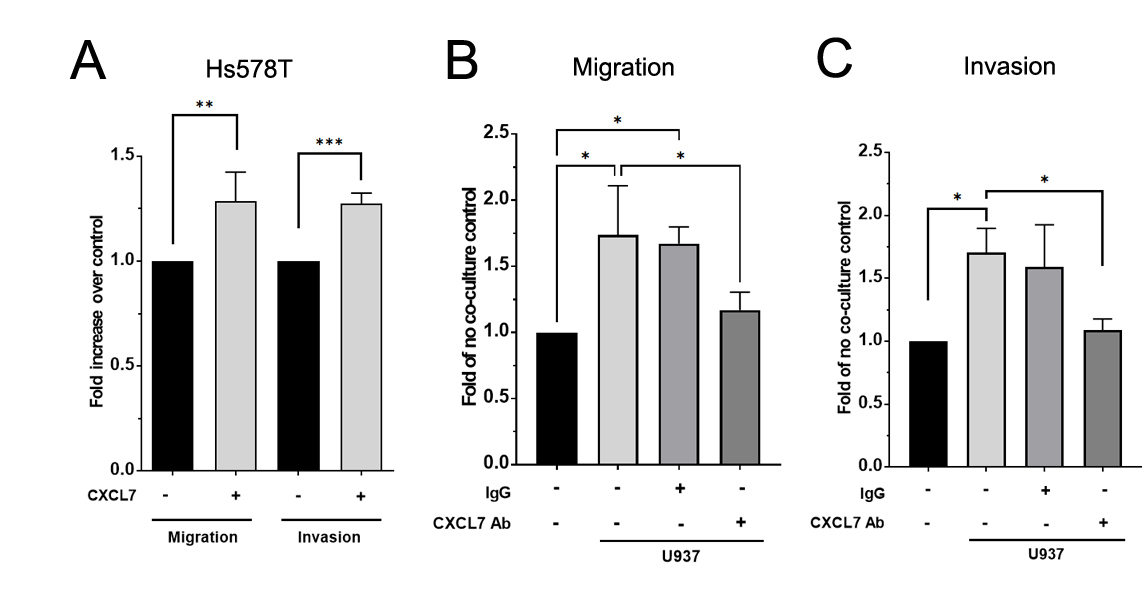
Supplementary Figure 3. CXCL7 induces breast cancer cell migration and invasion.**

(A) Migration and invasion abilities of Hs578T cells pre-treated with rCXCL7 (2 ng/ml) for 48 h. Data represent the normalized means ± SD (n=3 biological replicates; **, *p*<0.01; ***, *p*<0.001).

(B, C) Migration and invasion abilities of MDA-MB-231 cells co-cultured with U937 monocytes in the presence of a CXCL7 neutralizing antibody (5ug/ml) or a control IgG for 48 h. Cells were then subjected to migration (B) and invasion (C) assays. Data represent the normalized means $\pm$SD (n=3 biological replicates; *, *p*<0.05).

**
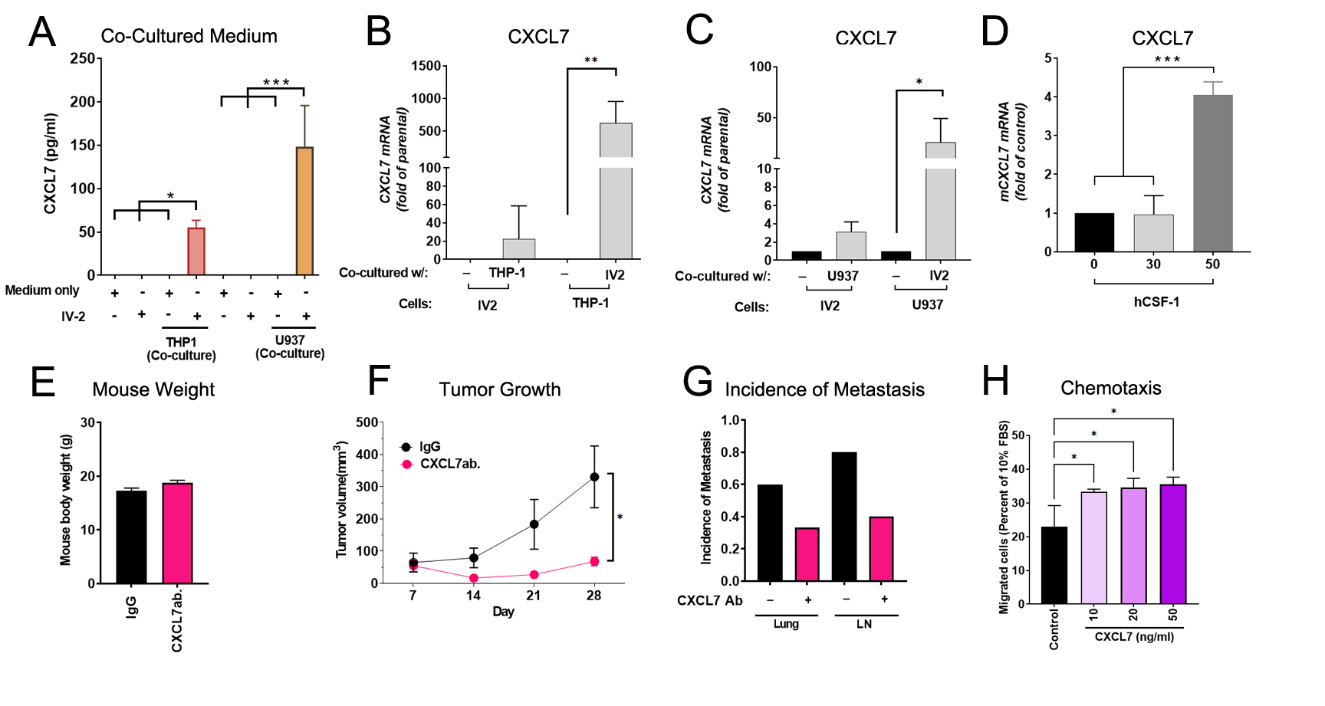
Supplementary Figure 4. Intravenous injection of a CXCL7 neutralizing antibody suppresses tumor growth and distant metastasis in a mouse xenograft model.**

(A) The CXCL7 protein concentration of cultured media from medium only, MDA-MB-231-IV2 (IV2), THP-1, and U937 cells alone, or from IV2 cells co-cultured with THP-1 or U937 cells for 48 h were determined by ELISA assay. Data represent the means$\pm$SD (n=3 biological replicates; *, *p*<0.05 ***, *p*<0.001).

(B, C) CXCL7 mRNA expression in MDA-MB-231-IV2 cells co-cultured with THP-1 (B) or U937 (C) cells for 48 h. Data represent the normalized means $\pm$SD (n= 3 biological replicates, *, *p*<0.05; **, *p*<0.01).

(D) Mouse CXCL7 (mCXCL7) mRNA expression in primary mouse monocytes treated with recombinant human CSF1 (30, 50 ng/ml) for 48 h, RNA was extracted to detect mCXCL7 expression. Data represent the normalized means $\pm$SD (n=3 biological replicates; ***, *p*<0.001)

(E-G) 2 x 10^6^ IV2 cells were orthotopically injected into the 4th mammary fat pads. Seven days after implantation, intravenous injection of a CXCL7 antibody (5mg/kg) or equivalent amount of control IgG_1_ was started once a week until day 28. The mouse weight (E) and the tumor growth curve (F). Data are means ± SEM (n=3-5). Two-way ANOVA was used for the statistical analysis (*p*<0.05). (G) Incidence of cancer metastasis in lung and axillary lymph nodes (LN). The incidence of distant metastasis was determined by human GAPDH mRNA detection via real-time RT-PCR.

(H) Chemotaxis assay in response to different doses of recombinant CXCL7 (10-30 ng/mL) for 16 hours. The percentage of migrated THP-1 cells (initial plating number: 5 x 10^5^) were calculated by the fluorescent intensity, and cells migrated toward medium containing 10% FBS are considered as 100 percent migration. Data represent percentage of migrated cells $\pm$SD (n= 3 biological replicates, *, *p*<0.05).


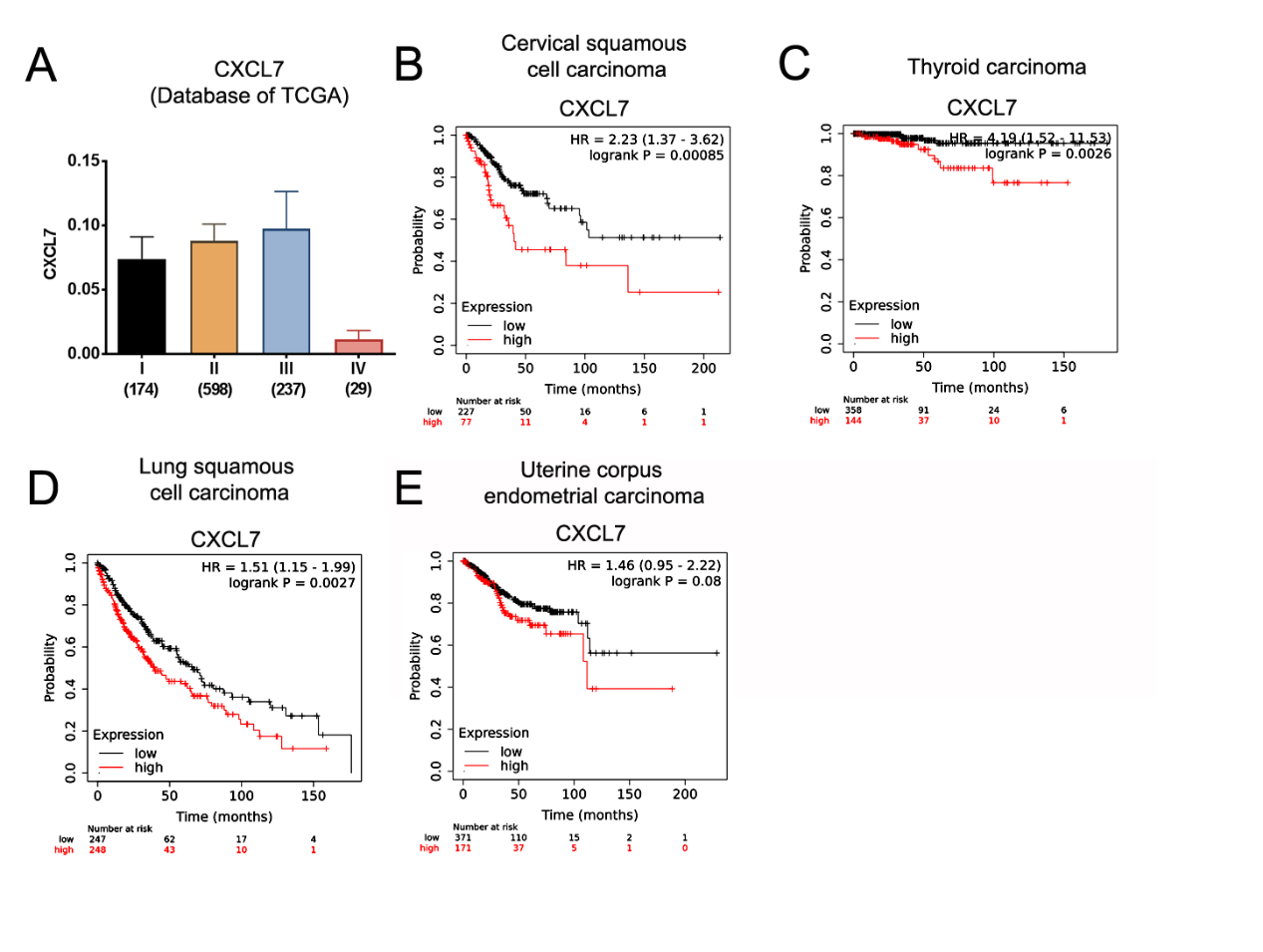


**Supplementary Figure 5. The mRNA expression of CXCL7 in TCGA-BRCA dataset and survival correlation in different cancer types.**

(A) The mRNA expression of CXCL7 in BRCA specimens obtained from TCGA. The case number of each stage is indicated in the bottom of the graph. Data represent the means ± SEM.

(B-E) Kaplan-Meier plots showing overall survival (OS) in Cervical squamous cell carcinoma (B), Thyroid carcinoma (C), Lung squamous cell carcinoma (D), and Uterine corpus endometrial carcinoma (E) and its association with CXCL7 expression from KM plotter RNA-seq datasets. The statistical significance was determined using the χ2 test.

**Supplemental Table**

| **Gene Name** | **5’ →3’** |
| --- | --- |
| siFAK | CCCAGGUUUACUGAACUUAtt |

**Supplemental table S1. Sequences of small interfering RNA used in this study.**

| **Recombinant protein** | **Cat. No.** | **Brand** |
| --- | --- | --- |
| hM-CSF | 216-MC | R&D systems |
| CCL2 | 279-MC | R&D systems |
| hCXCL7 | 393-NP | R&D systems |
|  |  |  |
| **Antibody** | **Cat. No.** | **Brand** |
| F4/80 | Ab6640 | Abcam |
| CD206 | 17-2069 | eBioscience |
| CXCL7 | MAB393 | R&D systems |
| M-CSF | sc-13103 | Santa Cruz Bio |
| Mouse IgG | 550854 | BD Pharmingen |
| FAK | 05-537 | Merck Millipore |
| FAK (Y861) | 07-832 | Merck Millipore |
| MMP13 | MAB3321 | Merck Millipore |
| GAPDH | sc-32233 | Santa Cruz Bio |
| IgG_1_ isotype control  (For *in vivo* experiment) | MAB002 | R&D systems |
| IgG mixture control  (For other experiments) | I5381 | Sigma-Aldrich |

**Supplemental table S2. Antibodies and recombinant proteins used in this study.**

| **Gene name** | **5’ →3’** |
| --- | --- |
| hCXCL7 | GCAACCAAGTCGAAGTGATAGCCA  ATCAGCAGATTCATCACCTGCCAA’ |
| mCXCL7 | TGGGCCTGATCCTTGTTGCGC  GCACCGTTTTTTGTCCATTCTTCAG |
| hCSF1 | GGGAATTCTAAGCTGGACGCAC  GTCTGCGGGTTGAGACAGAG |
| GAPDH | CCTCAAGATCATCAGCAATG  GTCCTTCCACGATACCAA |
| hGAPDH | TCCTGGTATGACAACGAAT  GGTCTCTCTCTTCCTCTTG |
| Actin | CGGCATCGTCACCAACTG  TCTCAAACATGATCTGGGTCATCT |

**Supplemental table S3. Primers used in this study.**
